# Progressive changes in binocular perception from stereopsis to rivalry

**DOI:** 10.3389/fpsyg.2024.1472278

**Published:** 2024-11-11

**Authors:** Yohske Hasegawa, Hirohito M. Kondo

**Affiliations:** ^1^School of Psychology, Chukyo University, Nagoya, Japan; ^2^School of Contemporary Sociology, Chukyo University, Toyota, Japan

**Keywords:** stereopsis, binocular rivalry, ambiguous perception, disparity, monocular image

## Abstract

**Introduction:**

The binocular system provides a stereoscopic view from slightly different retinal images and produces perceptual alternations, namely, binocular rivalry, from significantly different retinal images. When we observe a stereogram in which the stimulus configurations induce stereopsis and rivalry simultaneously, the binocular system prefers stereopsis to rivalry. However, changes in visual perception are yet to be investigated by parametrically manipulating the components of a stereogram.

**Methods:**

We examined stereopsis preferences in stereograms with various horizontal disparities. The stereograms of our paradigms included horizontal and vertical bars in one eye and a vertical bar alone in the other. Under experimental conditions, the vertical bar superimposed on the horizontal bar varied its position relative to the opposite vertical bar: range of horizontal disparity, 0.0′ to 42.3′. The superimposed vertical bar was absent under the control condition. Observers were instructed to indicate the disappearance of monocular horizontal bars, that is, targets, from their perception during the 30-s trials.

**Results:**

The total disappearance duration decreased under experimental conditions compared with that under control conditions, and it gradually increased with an increase in the disparity of the stereoscopic vertical bars.

**Discussion:**

These results indicate that the disparity in the stereoscopic components biases binocular perception away from the rivalry between the vertical and horizontal bars toward the stereopsis of the vertical bars. Furthermore, the disappearance duration showed a unimodal and asymmetric distribution across all disparity conditions. This suggests that rivalry processing occurs in parallel when stereopsis is dominant. We found that stereopsis preference is an outcome of binocular perception selection biased by disparity.

## 1 Introduction

The visual system provides coherent depth perception from retinal images obtained from two horizontally separated eyes. This perception requires a binocular stereopsis process that infers 3D structures from disparities and an integration process that merges slightly different retinal images (Julesz, 1971; Ono and Barbeito, 1982). However, when different images are presented to each eye, observers perceive alternations in monocular views, that is, binocular rivalry (Alais and Blake, 2005; Kaufman, 1963). Binocular rivalry is characterized by suppression of the image of one eye, with the observer perceiving only the image of the other eye. Stereopsis and rivalry are contrasted in terms of integration or competition between different images presented to the left and right eye.

Stereopsis and rivalry are essential phenomena in the binocular system (Blake and Wilson, 2011; Wang et al., 2020; Wilson, 2017). They do not occur simultaneously in the dichoptic observation of a stereogram in which the left and right images are of the same stimulus but one superimposed a different image (Blake and Boothroyd, 1985; Blake et al., 1980; Harrad et al., 1994; Hochberg, 1964; Julesz and Miller, 1975). For example, in observing a vertical-grating stereogram with a superimposed horizontal-grating superimposed in one eye, the horizontal-grating was prevented from rivalry (Blake and Boothroyd, 1985). The observer can view binocular vertical grating and monocular horizontal grating for most of the time. In another example, a horizontal-grating was superimposed on one of the dichoptic circles with a horizontal disparity of 1°; the depth of the circle was hardly perceived by the observer as the incongruent grating suppressed one of the dichoptic circles (Hochberg, 1964). These contradictory findings demonstrate the antagonism between stereopsis and rivalry in binocular perception. The visual system delivers visual perception in response to certain stimuli (Leopold and Logothetis, 1999). In the case of antagonism, perception is chosen from stereoscopic vision or a view of alternating monocular images. Antagonism seems to reflect a perception of ambiguity.

The choice of the percept state is biased by stimulus parameters (Brascamp et al., 2015; Hupé and Rubin, 2003; Hupé et al., 2019; Levelt, 1965). In antagonism, stereopsis is generally perceived dominantly, reducing the perceived proportion of rivalry (Blake and Boothroyd, 1985; Blake et al., 1991). This stereoscopic preference may be related to changes in binocular perception depending on the disparity of a single binocular stimulus (Ono et al., 1977). Ono et al. (1977) manipulated the disparity of 2-degree diameter disks presented to both eyes and measured the perceived position of the disks over 30 s. For 0′, 15′, or 30′ disparities, the disks were more likely to fuse and be perceived in the central position. However, beyond a disparity of 45′, the proportion of central percepts decreased, and at disparities exceeding 60′, the disks were rarely perceived in the center. With increasing disparity, the proportion of non-central percepts and diplopia increased, but the proportion of non-central percepts remained at approximately 30% after 45′. Non-central percepts of the disks were attributed to rivalry suppression of one of the disks. As indicated by this change in percept proportions, the perception of a single binocular stimulus exhibits a disparity dependent trilemma (Erkelens, 1988; Riesen et al., 2019). Increased disparity induces stereopsis dysfunction as well as a reduction in the rate of fusion percept: isolation of fused images (Sohmiya and Sohmiya, 1990), prolonged stereopsis latency in random dot stereogram (Goryo and Kikuchi, 1971), and reduced accuracy of depth estimation (Ogle, 1952; Ritter, 1979). This dysfunction may reduce stereoscopic preference in antagonism.

Whether temporal characteristics of rivalry exist during antagonism is yet to be investigated. Rivalry has temporal characteristics with the distribution of the disappearance durations of the monocular image following a gamma or log-normal distribution. The fraction of disappearance durations does not predict the durations of other subsequent fractions (Fox and Herrmann, 1967; Hupé and Rubin, 2003; Levelt, 1967). If rivalry maintains these characteristics during antagonism, the process of rival components would continue unconsciously in parallel with the stereoscopic process.

**Figure 1:**
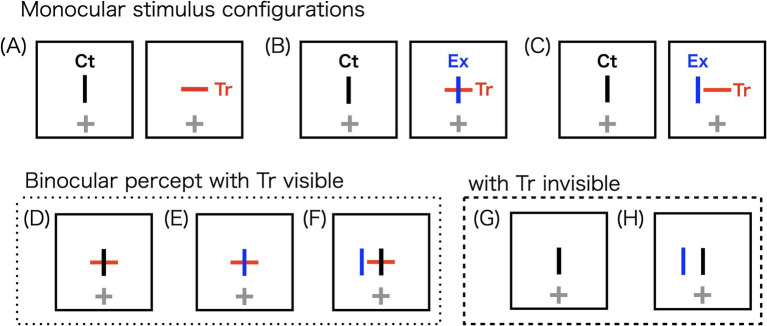
Schematic illustrations of the stereograms used in the experiment (A–C) and their potential binocular percepts (D–H). The horizontal bar represents the target (Tr), used to measure disappearance durations of the monocular image. Due to the orientation mismatch between Tr and the vertical control bar (Ct), the two bars enter into rivalry. The extra vertical bar (Ex), superimposed on Tr, generates a stereoscopic perception with Ct. Crosshairs indicate the fixation points. In the actual experiment, Ct and Ex were both presented in black, whereas fixation points were presented in blue. (A) A rivalry stereogram with orthogonal bars presented to each eye, forming a single-layer stereogram. (B) A 1.5-layer stereogram, which potentially produces stereopsis of the Ct-Ex combination and rivalry of the Ct-Tr combination. Fusion of the Ct-Ex combination occurs preferentially, reducing the likelihood of monocular target Tr disappearing. (C) Ex is horizontally displaced from Ct, while the Ct-Tr combination remains constant. (D) A stereoscopic percept in which Ct and Ex fuse, leaving Tr visible. (E) A potential rivalry outcome in which both Ex and Tr dominate the percept. (F) Diplopia, with no interaction between the bars. (G) A rivalry percept in which Ct dominates, causing the disappearance of both Ex and Tr. (H) A rivalry outcome in which Tr disappears.

In this study, we aimed to investigate stereopsis preference over rivalry using stereoscopic vertical bars with different horizontal disparities. In stereograms in which antagonism exists between stereopsis and rivalry, stereopsis is generally preferred (Blake, 1989; Blake and Boothroyd, 1985; Blake et al., 1991). We demonstrated that this stereopsis preference is constrained by disparity. Stereograms comprising vertical and horizontal bars were used to induce percepts of stereopsis or rivalry at the coincident retinal locations. The image presented to one eye contained only a vertical bar, while the image presented to the other eye contained a cross (Figure 1B). With one or two components in each eye, we referred to these stereograms as “1.5-layer” conditions. The horizontal bar (Tr) of the image with the cross on it served as the target for measuring disappearance duration. Since Tr disappearances are influenced by the stereopsis preference, the extra vertical bar (Ex) shortened the cumulative disappearance duration compared to a single-layer rivalry stereogram (Figure 1A). The single-layer stereogram consisted of an interocular pair of orthogonal bars: the vertical control bar (Ct) and Tr. Therefore, the difference in cumulative disappearance duration between the 1.5-layer and single-layer stereograms reflects a preference for stereopsis over rivalry. If the interference decreases as the horizontal disparity between Ct and Ex increases, this suggests a gradual transition from stereopsis to rivalry in the binocular system.

## 2 Experiment 1

### 2.1 Materials and methods

#### 2.1.1 Ethics statement

This study, involving human participants, was reviewed and approved by the Research Ethics Committee of Chukyo University (approval no. 2022–045). The experimental procedures were implemented in accordance with the Ethical Guidelines for Medical and Biological Research Involving Human Subjects. Written informed consent was obtained from all participants prior to their participation.

#### 2.1.2 Observers

Nine observers (four women and five men, aged 21–28 years, with one observer aged 69) participated in the experiment. Seven Observers were unaware of the purpose of the experiment. All observers had normal or corrected visual acuity and stereoacuity during dichoptic viewing, which was confirmed prior to the experiment. Stereoacuity was assessed using the Frisby Stereo Test (Clement Clarke International, Wales, UK). According to a power analysis, at least seven observers were required to detect a medium effect size (one-way analysis of variance [ANOVA]; *f* = 0.50, *α*-level = 0.05, 1 – *β* = 0.80).

#### 2.1.3 Stimuli

The 1.5-layer stereograms were designed for antagonism with the stereoscopic and rival components. The image in one eye had a horizontal target bar (Tr) and a vertical extra bar (Ex), while the image in the other eye had only a vertical control bar (Ct; Figures 1B,C). The stereogram was grey and spanned 5° on each side, with the bar at the center of the stereogram. Tr as the target was marked in red, with a length of 47′, width of 3.6′, and luminance of 2.58 cd/m^2^. The stereoscopic components, Ct-Ex, were black with a length of 47′, width of 3.6′, and luminance of 2.04 cd/m^2^. Small bars indicate exclusive suppression. Smaller images are unlikely to induce piecemeal rivalry, in which left- and right-eye images are perceived as a patchwork (Blake et al., 1992; Wilson et al., 2001). In a preliminary experiment, we found no Troxler effect for a monocularly presented target (Troxler, 1804).

The presentation location of Ex varied with the following conditions: five crossed horizontal disparities of 0.0′, 10.8′, 21.6′, 32.4′, and 43.2′ to Ct of the contralateral eye. The rival components, Ct-Tr, were invariant between the eyes, regardless of disparity.

In addition to the 1.5-layer stereograms, a single-layer stereogram was designed for rivalry with Ct-Tr. It comprised Tr in one image and Ct in the other. Dichoptic viewing with mismatched orientation features results in rivalry. Antagonism did not occur in the single-layer stereogram without stereoscopic components. The attributes of each stimulus were identical to those of the 1.5-layer stereograms.

Eye dominance influences the percentage of monocular image suppression in rivalry (Holopigian, 1989). The observers always viewed the target in the dominant eye to avoid measurement bias due to eye dominance. Eight observers were right eye dominant, with dominance checked using the hole-in-the-card test. Observers were instructed to look at a target six meters away with both eyes through a card with a hole in the center. When asked to close each eye alternately, the eye that could still see the target unobstructed by the card was identified as the dominant eye.

#### 2.1.4 Apparatus

The stimuli were displayed on a Samsung LED monitor s23b550v (23 inches). The refresh rate was 60 Hz, and the spatial resolution was 1,920 × 1,080. The observers viewed the stereograms on the monitor using a Sokkia Topcon mirror stereoscope MS16. The observers were seated in a dimly lit room with their chins on a chinrest. The viewing distance was approximately 57 cm. The observers indicated the invisibility of the targets using a keyboard. We conducted experiments using an Apple Mac Pro computer (mid-2012) running Python 3 to control the experiment and recorded the data.

#### 2.1.5 Task procedures

Each trial comprised three phases: preparation, observation, and rest. The stimulus presentation area and fixation points were displayed at the beginning of the trial. The observers moved on to the next phase by pressing the start key on the keyboard. A stereogram with a fixation point was presented for 30 s for the observation phase. A 15-s rest followed the observation phase to reduce the after-effects of the observation. When a beep signaled the end of the rest phase, the observers returned to the preparation phase. The preparation phase continued until the start key was pressed; this interval allowed the observers to take a break of 15 s or more at will.

The observers viewed the stereograms using a stereoscope in the order described above. Their task was to hold the response key during the observation phase while the target disappeared from view (Figure 1G,H). They were instructed not to hold the button down while the target was visible (Figure 1D–F). Visibility of the other bars and depth perception were not assessed. The observers were instructed to gaze at the fixation point during the observation and to refrain from blinking as much as possible.

We measured the duration of the disappearance of the target under five disparity conditions for the 1.5-layer stereograms and under one disparity condition for the single-layer stereograms. A total of 36 trials (six conditions × six repetitions) were conducted for each observer. The order of the trials was randomized among observers.

#### 2.1.6 Data analyses

**Figure 2:**
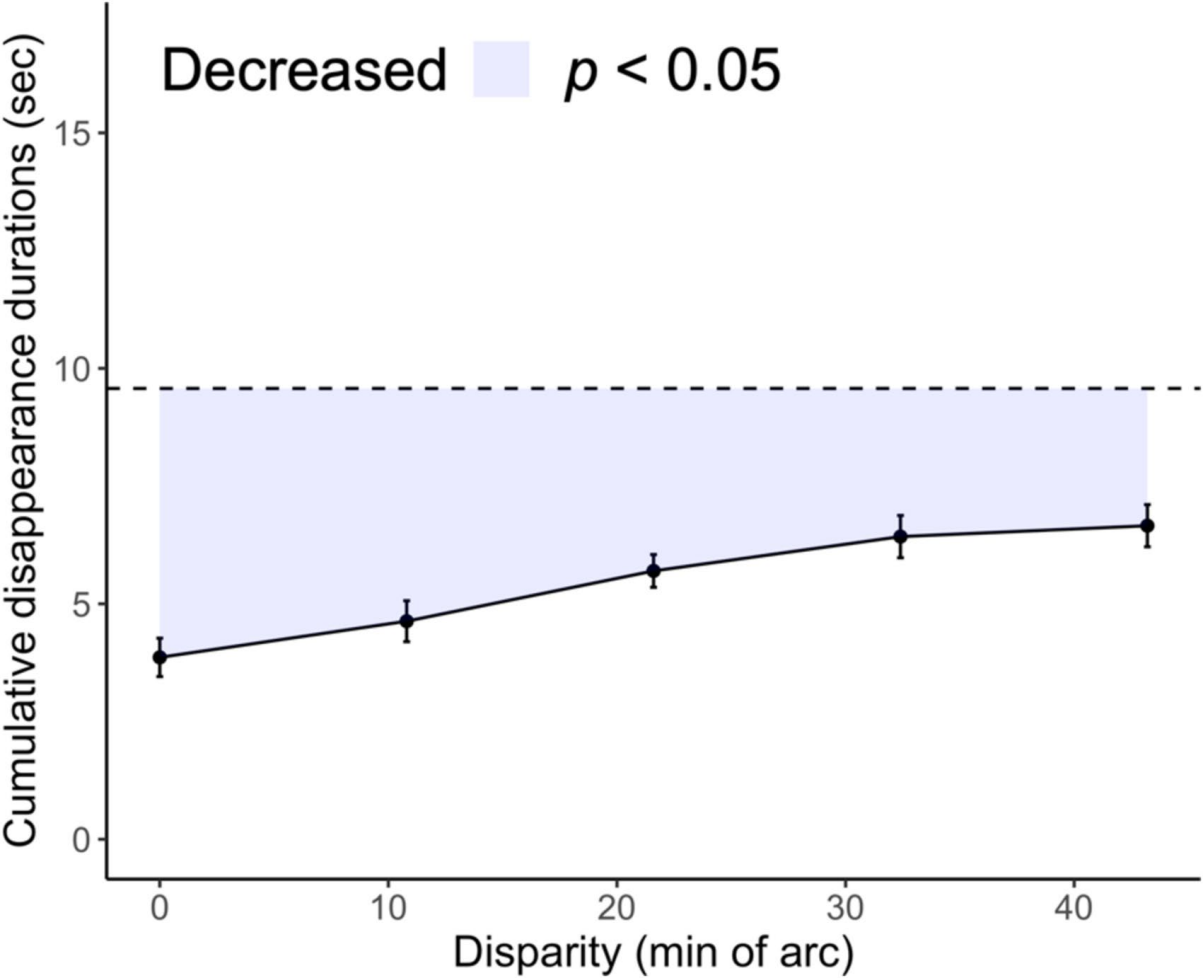
Comparison of cumulative target disappearance durations in 1.5- and single-layer stereograms. The cumulative disappearance durations for the 1.5-layer stereogram are a function of the horizontal disparity of the vertical bars. The colored area indicates interferences. Black dots indicate the group mean, whereas error bars represent the standard error. The dashed line indicates the mean cumulative disappearance duration of the single-layer condition for comparison.

**Figure 3:**
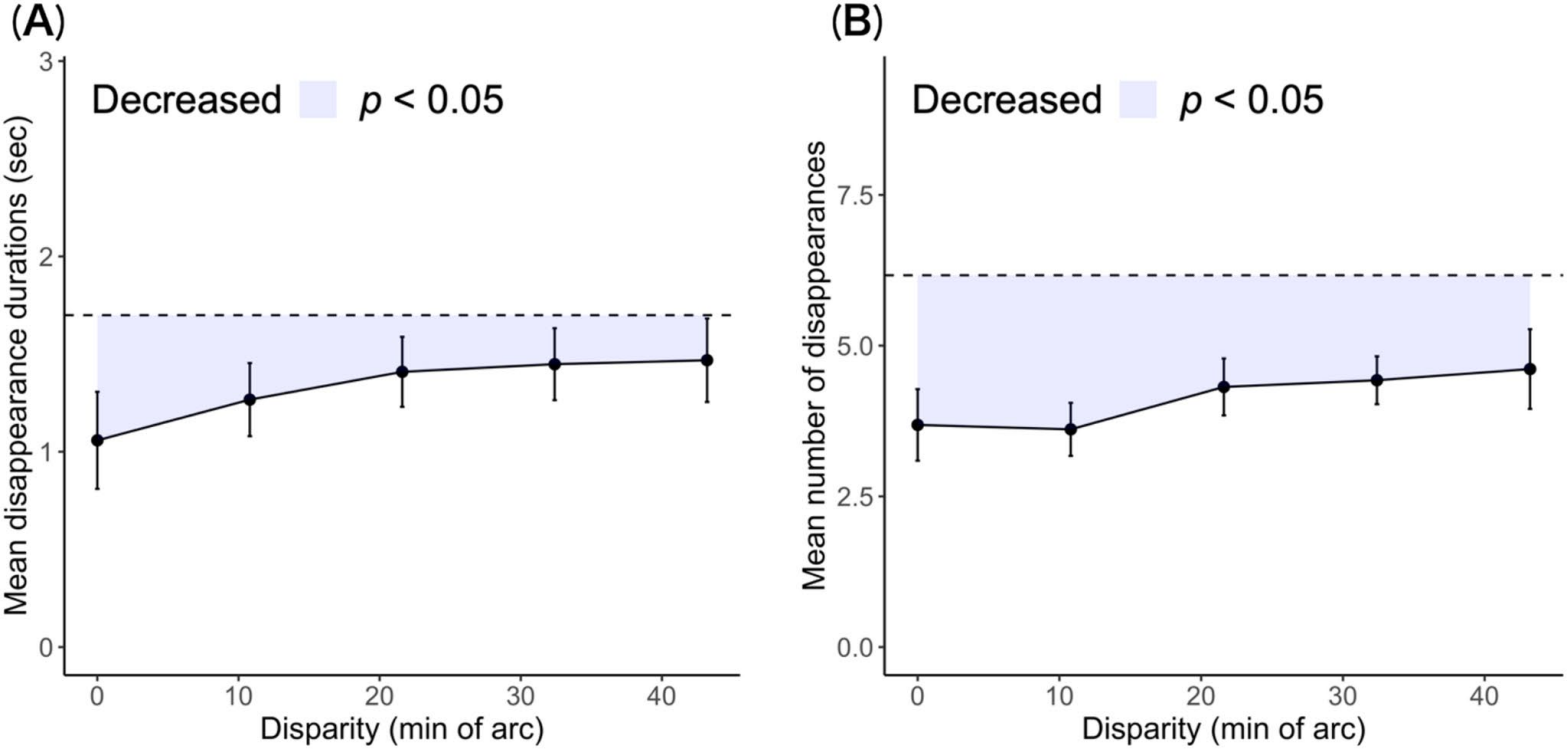
Mean disappearance durations and number of transitions are plotted as a function of horizontal disparity. (A) Disappearance durations averaged across observers. (B) Number of transitions per trial duration averaged across observers. The error bars are the standard errors. The dashed line indicates the mean disappearance durations and number of transitions under the single-layer condition for comparison. The colored area indicates interferences.

All analyses were performed using R version 4.1.2. We considered three dependent variables: the interferences of the cumulative disappearance durations, disappearance duration, and disappearance number. Interference was expressed as the difference between the total time spent holding down the key in the single-layer condition and in each of the 1.5-layer conditions. This indicated a stereopsis preference for rivalry without individual variation in the ease of monocular disappearance. Interferences are indicated using the colored areas in Figures 2, 3.

The Jonckheere–Terpstra tests (Jonckheere, 1954) were used to identify trends in the reduction of dependent variables. These tests determined whether the observed data could be monotonically increased or decreased along the order. Separately, a one-way ANOVA was performed to compare the interferences among the five disparity conditions. Multiple comparisons were corrected using Holm’s method.

The reported log-normal and gamma distribution fits were computed as maximum-likelihood estimations based on all conditions, with standardization across observers. We standardized the durations using the mean duration and standard deviation, ignoring the layer conditions. Standardized durations indicate distribution trends without the individual ease of monocular disappearance. The criterion for excluding outliers was standardized disappearance durations greater than 10 SD. One duration was excluded from the analysis. We performed Kolmogorov–Smirnov (KS) and Shapiro–Wilk (SW) normality tests to compare the disappearance duration distribution with the given distributions. The KS tests determined whether the observed durations were statistically distinct from the given distributions. SW tests were performed for normally distributed data.

An autocorrelation analysis of disappearance durations was performed for each observer. Trials with three or more responses were included in the calculation of the correlation coefficient.

### 2.2 Results and discussion

#### 2.2.1 Cumulative disappearance durations

The time required to hold down the key per trial was summarized. The cumulative duration of target disappearance indicates the rivalry suppression probability (Figure 2). For the single-layer stereogram, the cumulative disappearance duration (mean ± SD) was 9.57 ± 2.99 s at baseline. For 1.5-layer stereograms, the cumulative disappearance duration was 3.86 ± 2.56 s at the 0.0′ disparity level but increased to 6.66 ± 3.01 s at the 43.2′ level. The cumulative disappearance duration at the 0.0′ level was significantly lower than that for the single-layer stereogram: *t* (8) = 8.07, *p* < 0.001, Cohen’s *d* = 2.69. Furthermore, the cumulative disappearance duration was lower for any level of 1.5-layer stereograms than that for the single-layer stereogram. This reduction was attributed to the addition of Ex, which binocularly corresponded to Ct. This reduction supports previous findings (Blake and Boothroyd, 1985), which showed that fusion was preferred over rivalry when the stimuli were fusible in both eyes. However, our stereoscopic components did not completely dominate the rivalry because the cumulative disappearance duration at the 0.0′ level of the 1.5 layer stereogram was not zero: one-sample *t*-test: *t* (8) = 4.53, *p* = 0.002, Cohen’s *d* = 1.51.

Figure 2 shows the interference trend for each disparity condition. The interference tended to decrease with increasing disparity between the vertical bars of the left and right eyes: Jonckheere–Terpstra test, *JT* = 248, *p* = 0.001. This trend suggests that rivalry suppression of monocular images becomes frequent with the instability of stereopsis. We found a significant effect of disparity: *F* (4, 32) = 6.36, *p* < 0.001, *η*_p_^2^ = 0.442. The interferences at the 32.4′ and 43.2′ levels decreased compared with those at the 0.0′ level, *t*s (8) > 4.42, adjusted *p*s < 0.05.

#### 2.2.2 Distribution of the disappearance durations

**Figure 4:**
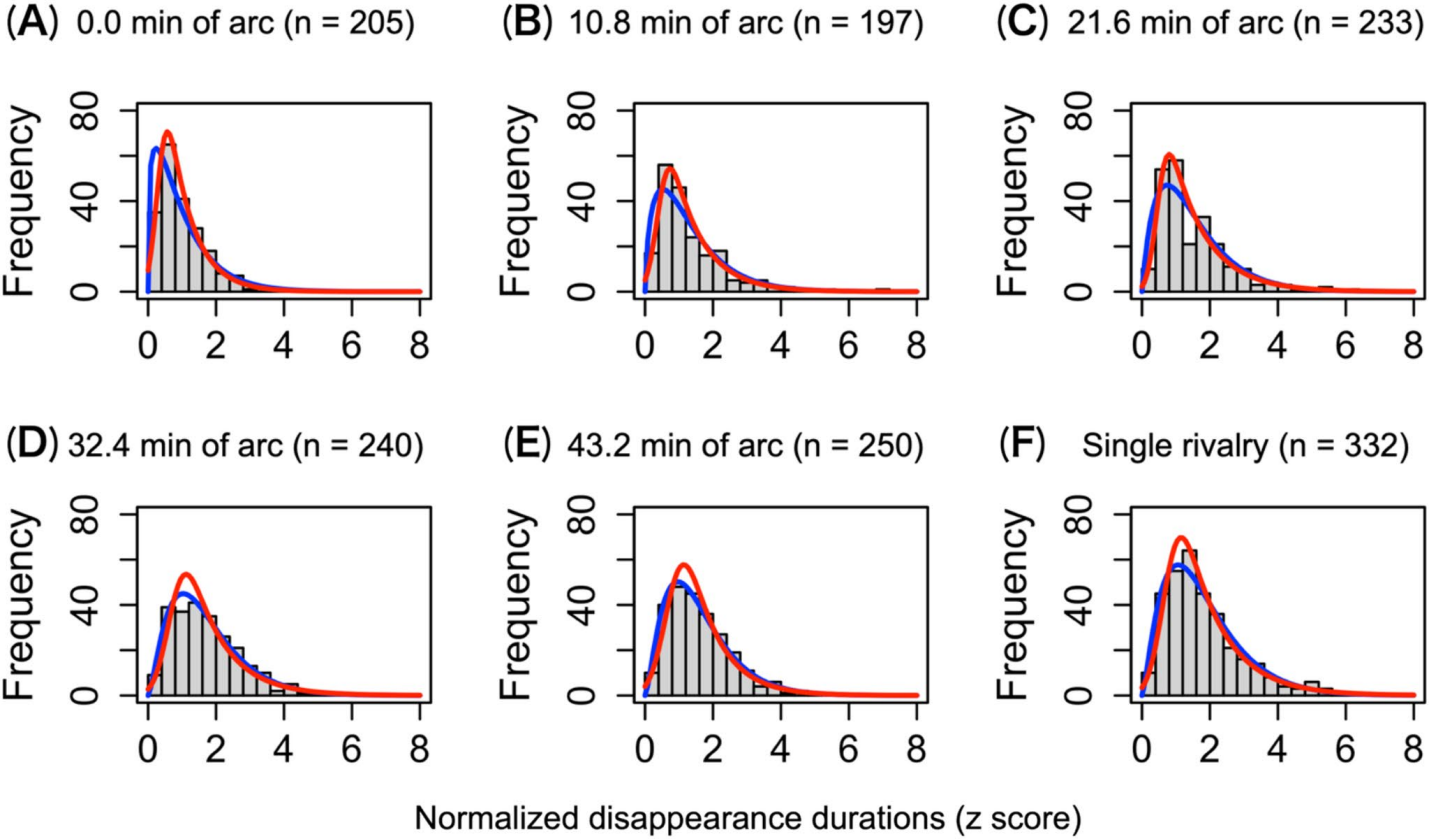
Histograms of the standardized disappearance durations across observers. (A–E) The disappearance duration distributions under each disparity condition in the 1.5-layer stereograms. (F) The disappearance duration distribution under the single-layer stereogram. The red and blue solid lines indicate log-normal and gamma fitting, respectively. n indicates the total frequency of each condition.

In rivalry, histograms of disappearance durations follow gamma or log-normal distributions (Hupé and Rubin, 2003; Kashino et al., 2007). We investigated whether the target disappearance durations for the 1.5-layer stereograms were distributed similarly to those in the single-layer rivalry. Figure 4 shows the distribution of the standardized disappearance duration for each condition. The overall mean disappearance duration was 1.36 ± 1.08 s.

The distribution of standardized disappearance duration did not follow a normal distribution but fitted well to the log-normal and gamma distributions. When comparing the Akaike information criterion, the log-normal distribution had a better fit than the gamma distribution under all conditions (Akaike, 1974). The KS test did not detect a difference in the shape of the distributions from the log-normal or gamma distributions, *p*s > 0.305 for the log-normal; *p*s > 0.087 for the gamma (except the gamma distribution in the 0.0′ condition, which has a steep slope, *p* = 0.005). The SW normality test revealed that none of the disappearance duration distributions was normal, *p*s < 0.001. The unimodal and asymmetric distribution of duration disappearance indicates that perceptual alternations, such as binocular rivalry, occur during antagonism. Comparing the distribution of disappearance durations for each level of 1.5-layer stereograms (Figures 4A–E) with that for single-layer stereograms (Figure 4F), we found that the shape of the distributions for 0.0′, 10.8′, and 21.6′ was different: KS test *p*s < 0.002 (Figures 4A–C). There were no differences in the shapes of the remaining levels (Figures 4D,E) and the single-layer stereogram. With increased disparity, the distribution of disappearance durations during antagonism was indistinguishable from the single-layer condition in which rivalry occurred alone. Therefore, the stereopsis preference was less active under the 32.4′ and 43.2′ conditions.

**Figure 5:**
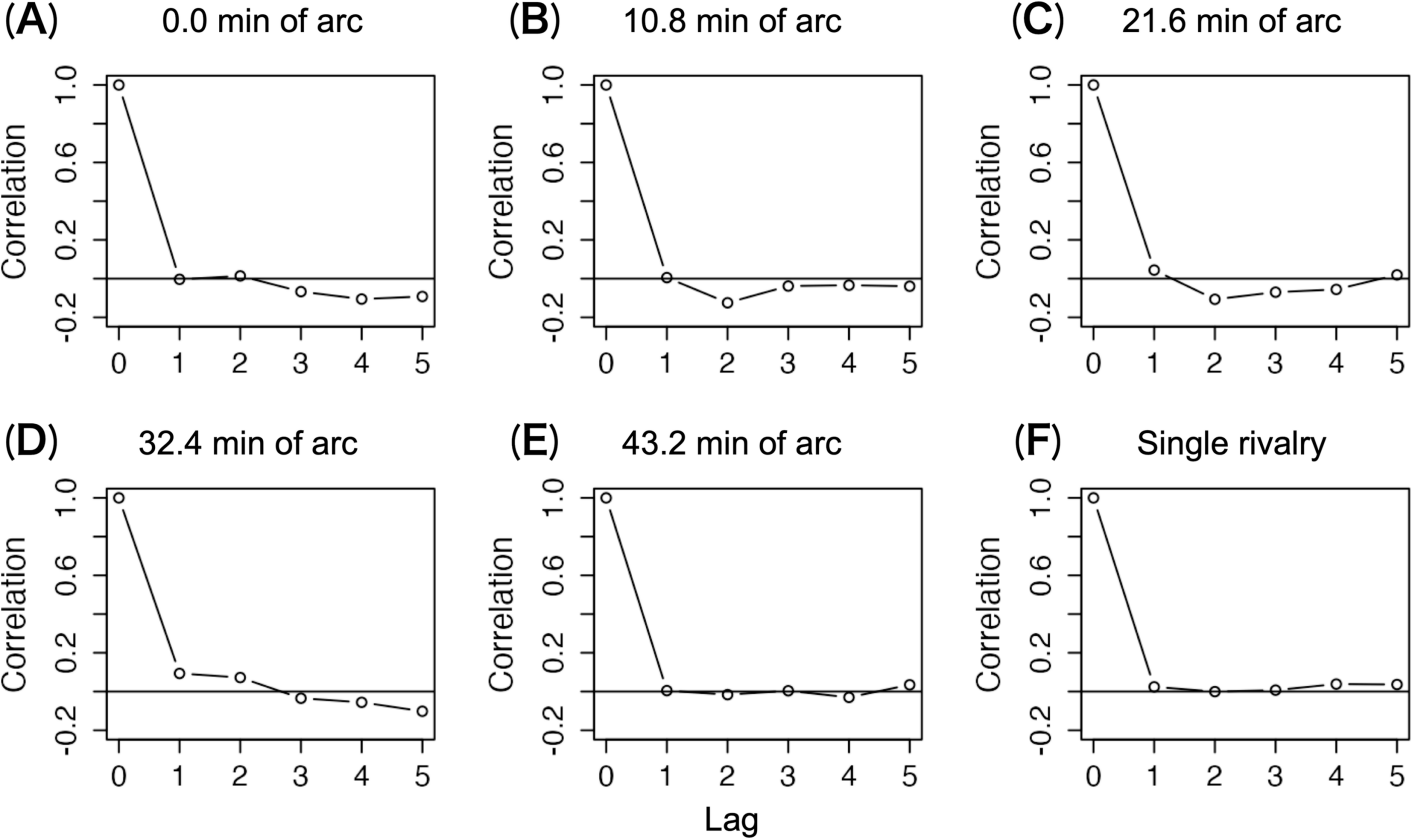
Autocorrelation functions for time series of disappearance durations. The mean of nine autocorrelations is shown. (A–E) The mean correlation coefficients under each disparity condition in the 1.5-layer stereograms. (F) The mean correlation coefficients under the single-layer stereogram.

Figure 5 shows mean Pearson correlation coefficients for nine observers. Correlations between disappearance durations were lacking under all conditions. This means that the preceding duration does not predict the following duration (Fox and Herrmann, 1967; Hupé and Rubin, 2003; Lehky, 1995; Levelt, 1967), even in the presence of stereoscopic components.

#### 2.2.3 Mean disappearance durations and number of disappearances

The reduction in the cumulative disappearance durations reflected a decrease in the mean duration and disappearance numbers. We examined whether this reduction was caused by one or both of these factors. The mean duration and number of disappearances for each disparity condition are shown in Figure 3.

The mean disappearance durations decreased in the range of 21.6′ to 0.0′ disparity levels, and the mean durations above the 21.6′ condition were similar to those of the single-layer stereogram. The mean disappearance durations in the 0.0′ condition were shorter than those in the single-layer condition: *t* (8) = 3.72, *p* = 0.006, Cohen’s *d* = 1.24. This shortening was caused by the additional bars. Next, we subtracted the durations of the disappearance of the 1.5-layer from single-layer stereograms for each observer to eliminate individual differences. These remainders were the quantity of stereoscopic interference at duration. The interference gradually faded as the disparity between the stereoscopic components increased: *JT* = 309, *p* < 0.027.

Targets can be invisible with rivalry suppression or visible without suppression. Here, state transitions from visible to invisible were counted as disappearances. The observers displayed fewer disappearances at the 0.0′ disparity level than in the single-layer condition: *t* (8) = 4.12, *p* = 0.003, Cohen’s *d* = 1.37. This difference reflects interference with rivalry, which affected the number of disappearances. Subsequently, we calculated the loss by differencing the number of disappearances of the 1.5-layer and single-layer for each observer. Disappearances were more frequent with increasing disparity in stereoscopic components: *JT* = 317, *p* = 0.037.

#### 2.2.4 Two interpretations of increased disappearance durations

The results of the current study show that the duration of target disappearance increases with the disparity of the stereoscopic component. Whether a single binocular stimulus is perceived as stereoscopic depends on its disparity (Goryo and Kikuchi, 1971; Ono et al., 1977; Riesen et al., 2019). In the case of the 1.5-layer stereogram, which contains both potential stereopsis and rivalry, Ct and Ex at large disparity levels are hardly perceived as stereoscopic and cannot interfere with rivalry between Ct and Tr.

However, our results can also be interpreted in terms of the modified version of Levelt’s law (Brascamp et al., 2015; Levelt, 1965). The antagonism between stereopsis and rivalry assumes that each stimulus is processed independently. However, if we consider Ex and Tr bars in one eye as a conjunct stimulus, their antagonism can be explained as single-layer rivalry in accordance with Levelt’s law. According to Levelt’s law, if the stimulus strength increases—due to factors such as mean luminance or spatial frequency—the conjunct stimulus becomes stronger relative to Ct in the other eye. The modified version of Levelt’s law, as outlined by Brascamp et al. (2015), can be summarized as follows:

1. Increasing the stimulus strength in one eye reduces cumulative disappearance durations of the stimulus in that eye.
2. Increasing differences in stimulus strength between the two eyes reduces mean disappearance duration of the stronger stimulus.
3. Increasing differences in stimulus strength between the two eyes reduces the number of disappearances.

Levelt’s original second law posits that when the stimulus strength in one eye is manipulated, dominance durations of the other fixed-strength eye change. However, Brascamp et al. (2015) noted that earlier experiments testing this law have often used high stimulus strength for the fixed-strength eye and, until recently, have rarely examined cases with low strength settings. Brascamp et al. (2006) manipulated the contrast in one eye while keeping the contrast in the fixed-strength eye constant across four different levels. They found that dominance durations varied with stronger stimuli and were not confined to the fixed-strength eye.

Disappearance durations at the 32.6′ and 43.2′ disparity levels, where Tr and Ex were isolated, were significantly longer than those at the 0.0 min level. It is unclear whether these longer disappearance durations were due to stereopsis preference or to an increase in stimulus strength associated with stimulus conjunction.

These findings raise concerns about the influence of stimulus conjunction on the proportion of binocular percepts. Some targets that shared the visual field with the stereoscopic component may have been excluded from disappearance. We therefore conducted a subsequent experiment to investigate whether this increase in disappearance durations was associated with the dissolution of stimulus conjunction.

## 3 Experiment 2

The influence of endpoints is recognized in binocular fusion. Moving the diplopic images of the left and right eye closer leads to fusion, while pulling the fused binocular image temporal ward breaks fusion. The disparity required to break fusion is greater than that required to achieve fusion from diplopia, a phenomenon known as fusion hysteresis. When a bar presented to one eye is replaced by a dot that coincides with the bar’s endpoint, the dot and bar exhibit a fusion hysteresis of 70% compared to the fusion of the full binocular bar (Sohmiya et al., 1999).

**Figure 6:**
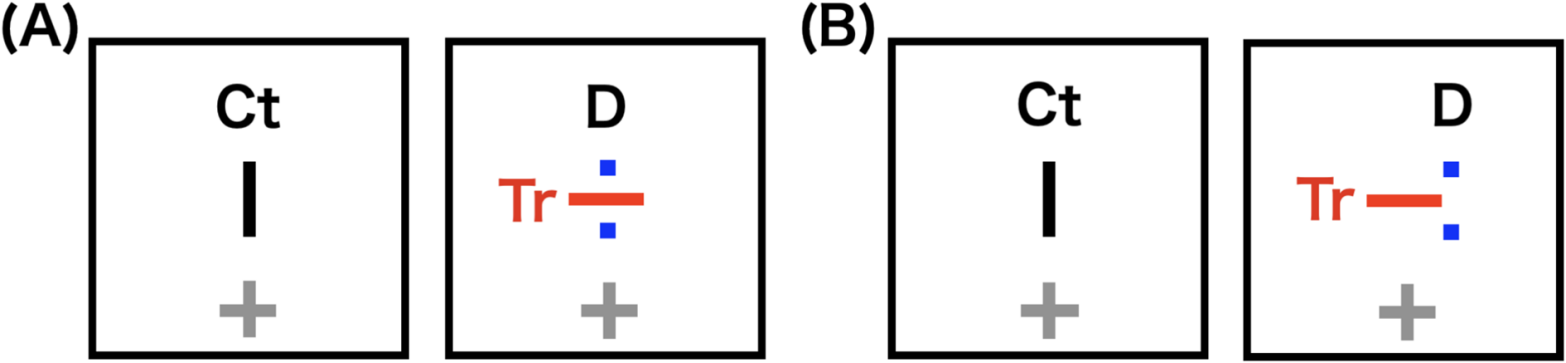
Schematic illustrations of stereograms used in the subsequent experiment. Potential percepts are either rivalry between control (Ct) and target (Tr) bars, or stereopsis of Ct and two dots (D). In the actual experiment, Ct and D were black. (A) 0.0′ horizontal disparity condition: Two dots (D) are positioned to coincide with the two endpoints of the bar Ct, with no disparity. (B) 43.2′ horizontal disparity condition: D are shifted horizontally from Ct, increasing the disparity.

We constructed a 1.5-layer stereogram by overlaying two dots D onto one image of the rivalry stereogram (Figure 6). These D were positioned to coincide with the two endpoints of the bar Ct presented to the opposite eye, and the disparity was manipulated by shifting D horizontally. This 1.5-layer stereogram produced an antagonism between stereopsis and rivalry without stimulus conjunctions between D and Tr. We measured the disappearance durations of horizontal bars Tr under various disparity conditions of Ct and D.

### 3.1 Materials and methods

Seven observers (two women and five men, aged 22–29 years) were recruited for Experiment 2. They had not participated in Experiment 1, and six were naïve to the purpose of the study. All observers had normal or corrected-to-normal visual acuity and stereoacuity.

Observers were instructed to fixate on a gaze point during the observation and to hold the left or up arrow keys when Tr or Ct disappeared, respectively. The time during which the keys were not held was the time during which the observer experienced stereoscopic perception.

A 1.5-layer stereogram variant of Experiment 1 was used, with Ex changed to D. D spanned to 3.6′ and were black with a luminance of 2.04 cd/m^2^. The vertical coordinates of D coincided with the endpoints of Ct, but the horizontal positions were manipulated as an independent variable: five crossed horizontal disparities of 0.0′, 10.8′, 21.6′, 32.4′, and 43.2′ relative to Ct in the contralateral eye. Tr and D were presented to the observer’s non-dominant eye; five were left-eyed non-dominant.

### 3.2 Results and discussion

**Figure 7:**
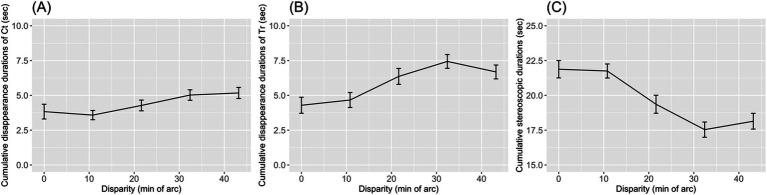
The mean cumulative disappearance durations of control (Ct) and target (Tr) bars were plotted to (A) and (B) as a function of the horizontal disparity of two dots. Error bars indicate standard errors of means. The mean cumulative stereopsis duration is shown in (C).

The cumulative disappearance durations of Ct and Tr were calculated by summing the response durations per trial. Figures 7A,B show that cumulative disappearance durations increased with the disparity of D. The cumulative disappearance duration of Tr was 4.29 ± 3.73 s at the 0.0′ disparity level, but it increased to 6.68 ± 3.23 s at the 43.2′ disparity level. Similarly, the disappearance duration of Ct was 3.83 ± 3.44 s at the 0.0′ disparity level, increasing to 5.17 ± 2.58 s at the 43.2′ disparity level. Both cumulative disappearance durations tended to increase with the disparity between Ct and D. For Tr, the Jonckheere–Terpstra test yielded *JT* = 307, *p* = 0.037, and for Ct, *JT* = 330, *p* = 0.008. This trend suggests that rivalry durations increase with the instability of stereopsis. The increase in the cumulative disappearance duration was greater for Tr than for Ct. This difference might be due to Tr being presented to the non-dominant eye.

The cumulative stereopsis duration was calculated as the non-response time per trial. Cumulative stereopsis duration decreased as the disappearance durations of Ct and Tr increased (Figure 7C), dropping from 21.88 ± 4.04 s in the 0.0′ disparity level to 18.14 ± 3.68 s at the 43.2′ disparity level. For the cumulative stereopsis duration, the Jonckheere–Terpstra test yielded *JT* = 144, *p* = 0.002.

There was no significant difference in Tr disappearance durations between the 0.0′ disparity level in Experiment 1 and the 0.0′ disparity level in the subsequent experiment: *t*(9.97) = 0.25, *p* = 0.805, Hedges’ *g* = 0.13. This suggests that stereopsis interference with rivalry is equivalent for both Ct-Ex combinations and Ct-D combinations.

## 4 General discussion

Our results demonstrate that stereopsis preference for antagonism with rivalry is constrained by horizontal disparities. Stereoscopic perception is usually preferred in 1.5-layer stereograms that include both stereoscopic and rival components (Blake and Boothroyd, 1985). However, we found that this preference was moderated when we manipulated the disparity of the stereoscopic components, which led to increased rivalry suppression, despite rival components remaining unchanged (Figures 2, 7). In experiments involving single-layer stereograms, the binocular system alternates between stereopsis or rivalry based on the disparity of binocular stimuli (Goryo and Kikuchi, 1971; Ono et al., 1977; Riesen et al., 2019). The limitation in stereopsis preference under antagonistic conditions can be attributed to the disruption of stereoscopic processing that is caused by the disparity dependence of binocular perception.

The preference for stereoscopic perception largely depends on the disparity in binocular stimuli. Our findings revealed a reduction in disappearance durations with stereopsis, whereas disappearance durations increased with increasing disparity in stereopsis components. This increase indicates that disparity plays a crucial role in modulating the degree of stereopsis preference. In most 1.5-layer scenes, stereopsis tends to be favored over rivalry due to the relatively small disparity. However, when the disparity of rival components is minimal compared to that of stereoscopic components, this preference is diminished. The modulation of preference by disparity is consistent with conflicting findings in previous studies, which suggest that either stereopsis or rivalry can prevail, depending on experimental conditions (Blake and Boothroyd, 1985; Hochberg, 1964).

The antagonism between stereopsis and rivalry is not characterized by a qualitative association but rather by a mutual dominance that varies with disparity. The reduction in the cumulative disappearance durations under the 1.5-layer conditions indicates that the presence of stereoscopic components interfered with the disappearance of rival components. This interference was evidenced by a shortening of the disappearance duration and a decrease in the frequency of disappearances (Figure 3). Despite this interference, rivalry suppression maintained the shape of log-normal distributions (Figure 4). The unimodal and asymmetric nature of disappearance durations reflects the stochastic nature of rivalry (Fox and Herrmann, 1967; Hupé and Rubin, 2003; Lehky, 1995; Levelt, 1967). The fact that these distributions were observed across most disparity conditions suggests that stereopsis attenuates rivalry phenomena rather than disrupting the processing of rivalry.

Disappearance durations among all disparity conditions followed a log-normal distribution, even when stereopsis was the preferred percept. It has been postulated that perceptual alternation arises from neural competition between visual inputs from each eye (Brascamp et al., 2006; Kalarickal and Marshall, 2000; Kang and Blake, 2010; Wilson, 2003). Essentially, neural representations of dominant percepts are experienced, but noise and adaptation lead to periodic alternations. The alternation timing is driven by noise, resulting in log-normal distributions of disappearance durations (Huguet et al., 2014; Moreno-Bote et al., 2007). In our 1.5-layer stereogram, two potential percepts could emerge after the disappearance of the target: either a stereoscopic view or a predominant perception of the target eye’s input due to rivalry. Conversely, only a single percept is possible with single-layer stereograms. If the timing of the alternation to the stereoscopic view is influenced by the stereopsis preference, one might expect a deviation from the log-normal distribution in disappearance durations. However, we found that the distribution remained log-normal, indicating that neural competition driving rivalry continued in parallel with stereopsis. This suggests that stereopsis preference may influence the ease of alternation without disrupting the rivalry process as a whole.

The perception of surfaces occluded by another surface is likely to be suppressed. This principle of occlusion is widely observed in visual disappearance phenomena such as binocular rivalry and motion-induced blindness (Graf et al., 2002; Shimojo and Nakayama, 1990). In our stereograms, the stereoscopic component in front may have suppressed the target in line with this occlusion principle. However, the increase in Ct’s disappearance duration in subsequent experiments suggests that the extended disappearance of Tr is due to reduced stereopsis preference rather than the occlusion principle. Despite the requirement for stable stereopsis for Ct to occlude Tr, Ct’s disappearance duration also increased under large disparity conditions. This tendency for both Ct and Tr to have longer disappearance durations reflects an overall increase in rivalry strength.

We conceptualized stereopsis preference as an aspect of stereo matching. The antagonism between potentially two depth planes has been observed in ambiguous random-dot stereograms (Julesz, 1971; Julesz and Chang, 1976; Julesz and Johnson, 1968; Samonds et al., 2017). The image presented to one eye contains a horizontally offset duplicate of superimposed random dots, and the image in the other eye is unduplicated, with a dot ratio of one to two. The typical offset width corresponds to the disparity, generating both crossed and uncrossed disparities between the duplicated and unduplicated stimuli. In ambiguous random-dot stereograms, observers perceive only one depth plane at a time in a dichoptic view, as the binocular system selects one of stereoscopic depths. The perceived depth alternates periodically. This ambiguity in depth perception is biased by a preference for small disparities and the extrapolation of surrounding depth cues. For instance, in ambiguous random-dot stereograms where the crossed disparity is smaller than the uncrossed disparity, the nearer depth plane appears more frequently. If the crossed disparity increases while the uncrossed disparity remains constant, depth perception durations in the uncrossed disparity increases. This behavior of the binocular system has been described by cooperative stereo algorithms (Goutcher and Mamassian, 2006; Marr and Poggio, 1976). Ambiguous random-dot stereograms can be categorized as 1.5-layer stereograms, in which one pattern in one eye corresponds to two patterns in the opposite eye. Our findings that manipulating the disparity of stereoscopic components modulated rivalry suppression, are analogous to the depth selection mechanism in ambiguous random-dot stereograms. In both cases, the binocular system selects which of the duplicated stimuli in one eye engages with the single stimulus in the other eye, and this selection is disparity dependent. The key distinction from ambiguous random-dot stereograms is that in our case, one paired component induces rivalry due to the disparity dependence of binocular perception. This resemblance suggests that stereo matching inherently involves rivalry (Bingushi and Yukumatsu, 2005; Hayashi et al., 2004).

Disparity dependence and antagonism can be differentiated based on whether target stimuli are directly or indirectly manipulated when measuring variations in percept durations. In single-layer stereograms, smaller disparities lead to a higher proportion of fusion and depth perception, whereas larger disparities result in a higher proportion of rivalry states (Blake, 1989; Blake et al., 1991; Buckthought et al., 2008; Erkelens, 1988; Gillam and Rogers, 1991; O’Shea, 1987; Riesen et al., 2019). Most previous studies have directly manipulated target stimuli, such as the orientation disparity of vertical gratings, to measure stereoscopic or rivalry durations. In contrast, our 1.5-layer stereogram experiment showed that manipulating stereoscopic components affected the durations of target disappearance, even though rivalry components remained constant (see also Blake and Boothroyd, 1985). The impact of this indirect manipulation suggests that information from each component is integrated at a higher level of processing (Bingushi and Yukumatsu, 2005; Marr, 1982; Marr and Poggio, 1976; Tanabe et al., 2011). In early processing stages, the trade-off between stereopsis and rivalry is governed by the disparity dependence of local stimulus pairs. At higher stages, perceptual states at each location are reconciled to ensure consistency across the visual field. We propose that disparity dependence operates at early stages, whereas antagonism occurs at higher levels within the stereo matching system.

Stereopsis preference does not eliminate the interference of rivalry on stereopsis. Our findings demonstrate that the proportion of rivalry in binocular percept of 1.5-layer stereograms varies with the disparity of stereoscopic components. Conversely, the proportion of stereopsis can also vary with the disparity of rivalry components. Hochberg (1964) measured depth perception durations of an 8° diameter circle with a 1° uncrossed disparity and found that durations of binocular circles with superimposed monocular horizontal gratings were shorter than those of binocular circles presented alone. Furthermore, Hochberg (1964) presented partial gratings that were either congruent or incongruent with the circle in the opposite eye. The congruent gratings strongly interfered with stereopsis, whereas the incongruent gratings did not. This study highlights the antagonism between stereopsis and rivalry in terms of depth perception duration. Antagonism is the perceptual occupation of stereopsis and the rivalry that occurs in 1.5-layer stereograms producing two types of binocular percept. Stereopsis preference is one aspect of antagonism.

In the context of antagonism in 1.5-layer stereograms, the relative ease with which stereopsis or rivalry occurs determines the dominant percept. Neuroplasticity and adaptation can modulate their perceptions, as both stereopsis and rivalry reflect neural activity in the visual cortex. For instance, repeated viewing of random-dot stereograms has been shown to reduce stereoscopic latency (Goryo and Kikuchi, 1971), suggesting that enhanced sensitivity to disparity detection may increase the prevalence of stereopsis in 1.5-layer stereograms. Conversely, prolonged viewing can induce contrast adaptation, which has been found to extend durations of rivalry suppression (Lehky, 1995). This increased ease of disappearance may, in turn, elevate the proportion of rivalry in 1.5-layer stereograms.

Blake and Boothroyd (1985) reported that in dichoptic observations of 1.5-layer stereograms, no disappearance of monocular images occurred. In contrast, our data indicated a slight disappearance even under the 1.5-layer condition with 0.0′ disparity. This discrepancy may be a result of differences in stimulus parameters, such as luminance of contours. The binocular system tends to favor continuous depth planes over isolated contours or disordered depth planes. This preference is facilitated by contours with consistent disparity, which cooperatively reinforce other disparity matches. As a result, stimuli with more contours forming a coherent depth plane are more likely to enhance this cooperative preference. In our study, the 1.5-layer stimuli, with some contours fused at the same disparity, likely generated more rivalry interference compared to those with fewer contours. This stereo-matching algorithm accounts for differences between previous findings and our results.

We emphasize that the modulation of stereopsis preference by horizontal disparity is an integral aspect of binocular processing. Binocular perception is influenced by various factors, including shapes of stimuli, contrasts, contexts, and potential attention (Alais and Blake, 1999; Liu et al., 1992; Lew et al., 2021; Paffen et al., 2006). Consequently, stereopsis preference should be modulated not only by horizontal disparity but also by these other factors. In our study, we measured interference while controlling for these factors; therefore, the magnitude of the effects of horizontal disparity remains uncertain relative to other influences. In the context of 1.5-layer stereograms with contrast differences between stereopsis and rivalry components, the impact of horizontal disparity may be negligible.

However, the binocular system that mediates rivalry and fusion is not completely understood. The perception that arises is linked to the neural activity, with competition among these activities serving as a putative site of rivalry. Since this competition occurs differently in the visual stream, a hierarchical neural model of rivalry is necessary (Blake and Logothetis, 2002; Freeman, 2005; Lehky, 1988; Leopold and Logothetis, 1996; Logothetis and Schall, 1989; Sheinberg and Logothetis, 1997; Tong et al., 1998, 2006; Wilson, 2003; Xu et al., 2016). In the early visual cortex, interocular and lateral inhibition contribute to low-level competition (Polonsky et al., 2000; Tong and Engel, 2001). This region integrates binocular information to estimate three-dimensional structures (Barlow et al., 1967; Hubel and Wiesel, 1970; Nelson, 1975; Ohzawa et al., 1990). Effective modeling of visual cortical operations should encompass both stereopsis and rivalry (Li et al., 2017; Said and Heeger, 2013; Wang et al., 2020; Wilson, 2017). Recent binocular models addressing stereopsis and rivalry have emphasized disparity dependence. Our findings contribute to extending these models by highlighting that stereopsis preference during antagonism adheres to disparity dependence. Notably, the incorporation of rivalry in the global modulation step of stereo matching—part of the stereopsis model—presents an intriguing area for further exploration.

In conclusion, our results reveal that stereopsis preference in stereopsis and rivalry in superimposed stereograms are constrained by increasing disparity in stereoscopic components. This perceptual bias resulting from disparity is similar to the established stereoscopic depth selection in ambiguous stereograms (Julesz, 1971). Furthermore, the preservation of temporal characteristics during rivalry suppression under varying disparity conditions suggests that rivalry processing occurs unconsciously during stereoscopic viewing. These results indicate that stereopsis preference emerges as a consequence of binocular perception, affected by disparity. We have demonstrated that features of stereopsis in single-layer stereograms influence perceptual choice in the context of antagonism. Investigating the interconnectedness of stereopsis and rivalry using 1.5-layer stereograms enhances our understanding of the binocular system.

## Data Availability

The raw data supporting the conclusions of this article will be made available by the authors, without undue reservation. Data presented in this study are available at https://github.com/senri0304/gradual_change_s2r.
